# Effect of multilayer structure on high-frequency properties of FeCo/(FeCo)_0.63_(SiO2)_0.37_ nanogranular films on flexible substrates

**DOI:** 10.1186/1556-276X-8-212

**Published:** 2013-05-04

**Authors:** Li Zhang, Sen Qin, Xing Wang, Jianliang Xie, Longjiang Deng

**Affiliations:** 1 Engineering Research Center of Electromagnetic Wave Absorbing Materials, Ministry of Education, Chengdu 610054, China; 2 State Key Laboratory of Electronic Thin Films and Integrated Devices, University of Electronic Science and Technology of China, Chengdu 610054, China

**Keywords:** Multilayer film, High-frequency property, Flexible substrate, Anisotropy field

## Abstract

The high-frequency properties of the FeCo-SiO_2_ monolayer nanogranular films and FeCo/(FeCo)_0.63_(SiO_2_)_0.37_ multilayer nanogranular films which were elaborated on flexible substrates by magnetron sputtering system were studied. Compared to the monolayer films with the same FeCo content, the multilayer structures comprised of FeCo/(FeCo)_0.63_(SiO_2_)_0.37_ exhibit more excellent properties that the real and imaginary parts of permeability, more than the double value of the monolayer, increase to 250 and 350, respectively. The variation was considered owing to the reduction of the anisotropy field.

## Background

With the rapid increase of demand for the devices used in microwave band, ferromagnetic thin films with the potential for excellent magnetic property in the GHz range, owing to their special structure characteristics and free from Snoek limitation, have been widely studied in recent years.

The basic requirements for magnetic films operated in high frequency are high permeability (*μ*) and high resistivity (*ρ*) in GHz range, and metal insulating films, especially Fe and Co based films, have enormous potential to achieve a high permeability, owing to their high saturation magnetization and suitable anisotropic field [1-3]. For the monolayer ferromagnetic films, it is promising to achieve high microwave permeability to increase film thickness. However, the negative influence, the serious skin effect and eddy current [4,5], and the obvious out-of-plane anisotropy in the high frequency, will block the increasing of the permeability, while the thin magnetic films, with specific multilayer structure design, can efficiently avoid the above negative effect and improve high-frequency properties by leading into different dielectric layers [6]. In this study, FeCo-SiO_2_ monolayer films and FeCo/(FeCo)_0.63_(SiO_2_)_0.37_ multilayer films were prepared by co-sputtering and tandem sputtering on flexible substrates, respectively, and in order to discuss the improvement of multilayer films, the high-frequency properties of both films whose FeCo content was about 72 at % were investigated.

## Methods

FeCo-SiO_2_ monolayer films and FeCo/(FeCo)_0.63_(SiO_2_)_0.37_ multilayer films were all elaborated on flexible substrates by magnetron sputtering system under external bias magnetic field. The flexible substrates of this experiment were 13-μm thick polyethylene terephthalate films. A RF magnetron system was used to sputter SiO_2_, while DC magnetron cathode was used for FeCo target. The base pressure before deposition was under 1 × 10^−7^ Torr, and the working pressure during deposition was 2.5 × 10^−3^ Torr. The difference between the monolayer films and the multilayer films was the sputtering process. The FeCo-SiO_2_ monolayer films (120 nm) were prepared by co-sputtering both targets all time. For the FeCo/(FeCo)_0.63_(SiO_2_)_0.37_ multilayer films that were prepared by tandem sputtering, FeCo alloy layers (10 nm) and FeCo-SiO_2_ layers (20 nm) were sputtered alternately by controlling the shutter in front of the Si target. The total thickness of the films was also 120 nm, and the thickness of each layer could be managed by the deposition time.

The structure of the films was investigated by X-ray diffraction (model Bede D1, Durham, England) and transmission electron microscopy (TEM). Saturation magnetization, coercivity, and in-plane magnetic antisotropy field Hk were measured by BHV-525 vibrating sample magnetometer (VSM, Riken Denshi Co., Ltd., Tokyo, Japan). The microstructures and chemical composition of the samples were analyzed using a field emission scanning electron microscope and energy-dispersive spectroscopy. Complex permeability *μ* was measured in the frequency range of 500 MHz to 8 GHz by coaxial technique. The details of the measurement were discussed before [7].

## Results and discussion

**Figure 1:**
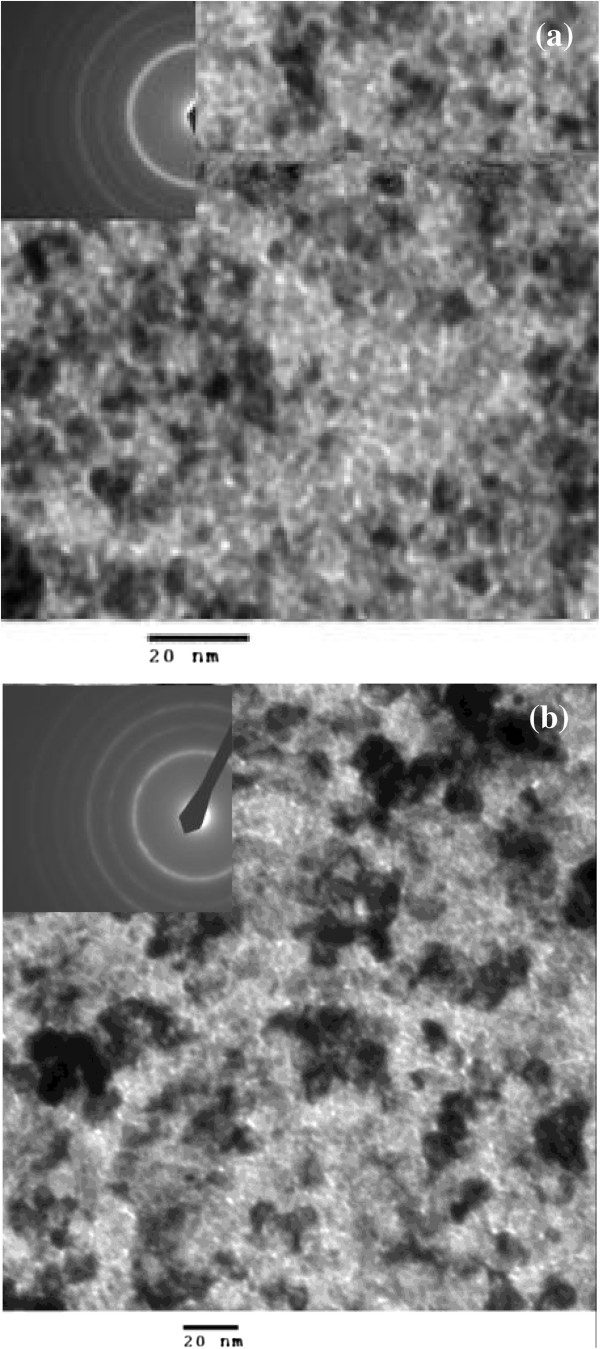
**Top-view TEM image and electron diffraction pattern of films: (a) FeCo-SiO**_**2**_**monolayer, (b) FeCo/(FeCo)**_**0.63**_**(SiO**_**2**_**)**_**0.37 **_**multilayer.**

**Figure 2:**
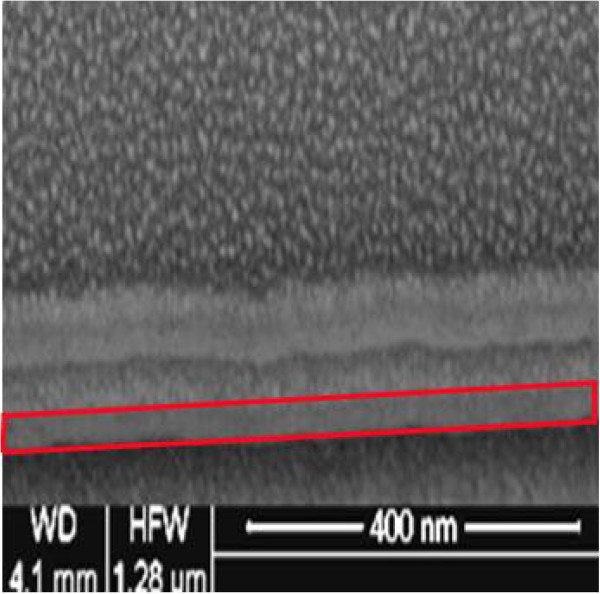
**Cross-sectional scanning electron microscopy (SEM) images of FeCo/(FeCo)**_**0.63**_**(SiO2)**_**0.37 **_**film.** Prepared by focused ion beam sectioning polished at 30 keV (the design thickness of the FeCo layer was 10 nm, and the FeCo-SiO_2_layer was 20 nm).

The top-view TEM image and electron diffraction pattern of the monolayer and multilayer films deposited on silicon nitride membrane window grids were shown in Figure 1. It was found that in both films, the FeCo metal particles were embedded in insulating SiO_2_ matrices and presented polycrystalline structure according to the electron diffraction patterns, and the FeCo particles size is about 5 to 7 nm. However, compared to the monolayer films shown in Figure 1a, the FeCo particles of multilayer films were reunited more observably in Figure 1b. The reason was analyzed and that the TEM shows all the information along the thickness direction which displays the particle information of the in-plane added in the FeCo layer. As the cross-sectional SEM image of multilayer films are shown on Figure 2, the total experiment thickness of a batch circled by red line, which includes a FeCo layer and a FeCo-SiO_2_ layer, was 30 nm. FeCo/FeCo-SiO_2_ interface in batches is difficult to discriminate. However, the phenomenon of the boundary between the batches was distinct and intuitively justifies the existence of the multilayer structure. The difference is considered as the influence of the compatibility.

**Figure 3:**
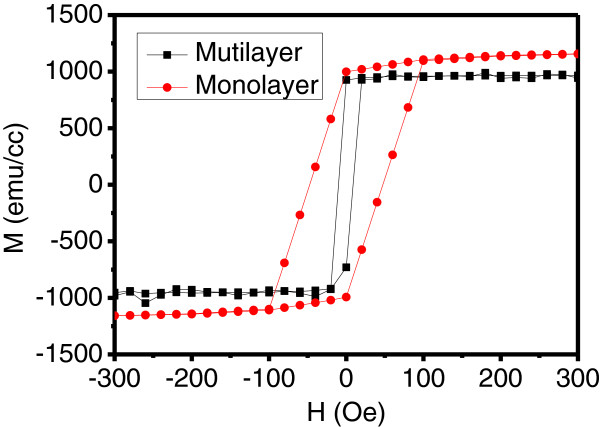
Hysteresis loops for monolayer and multilayer films.

The Hysteresis loops for monolayer and multilayer films were plotted in Figure 3, and the FeCo content of both films was about 72 at %. It was observed that the multilayer films had a much lower coercivity *H*_c_ about 10 Oe, while for the monolayer films, the coercivity was as high as 100 Oe. In our case, the change of the coercivity was the result of lower anisotropy field in multilayer films. Meanwhile for both films, the strait variation in the saturation magnetization which was decided by the content of magnetic phase was understandable.

**Figure 4:**
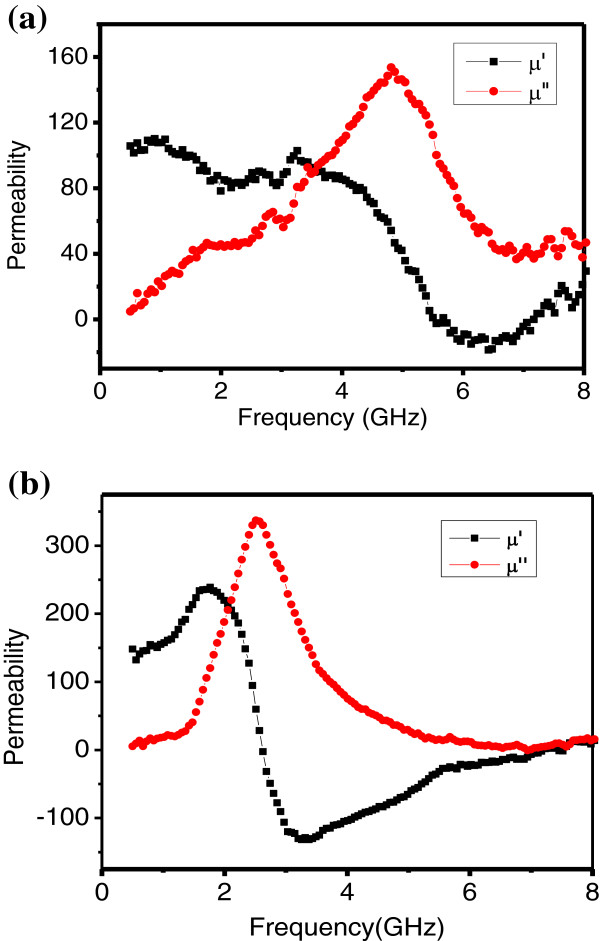
**The complex permeability of the films: (a) FeCo-SiO**_**2**_**monolayer, (b) FeCo/(FeCo)**_**0.63**_**(SiO**_**2**_**)**_**0.37 **_**multilayer.**

Then, contrasted to the high-frequency properties of the monolayer films (in Figure 4a) with the multilayer films (in Figure 4b), we can found that the complex permeability of the films which has multilayer structure had a huge improvement. The maximum real and imaginary parts of permeability, increasing twice higher than the monolayer films, were about 250 and 350, respectively, and a relatively wide frequency range that the imaginary part of permeability higher than 100 was from 1.7 to 4 GHz. However, the resonant frequency of multilayer films was decreased to 2.3 GHz simultaneously.

It is considered that for the monolayer structure FeCo-SiO_2_ films, almost the magnetism phase was isolated by non-magnetism phase because the FeCo particles were embedded in SiO_2_ matrices shown in Figure 1a. The magnetic structure of particles could be regarded as single domains due to the size of the magnetic particles smaller than the critical size of single domain which is dozens of nanometers for Fe_65_Co_3_[8]. Thus, the magnetic moment orientation of the single domain was their respective preferred direction and chaotic in plane, and the result relative to high in-plane anisotropy field of the films would improve the resonant frequency and coercivity and reduce the permeability.

Nevertheless, for the multilayer structure FeCo/(FeCo)_0.63_(SiO_2_)_0.37_ films, the domain orientation of individual FeCo layers was consistent owing to the applied magnetic field during sputtering. In order to certify the zero body magnetic charge and minimum magnetostatic energy, two adjacent FeCo layers presented reverse magnetic moment orientation. Meanwhile, the FeCo particles of FeCo-SiO_2_ layers which were similar to monolayer films could be regarded as single domain particles. However, not as chaotic of the magnetic moment in monolayer films, the magnetic moment orientation of FeCo-SiO_2_ layers was relatively consistent, which due to the exchange effect and also reported in the Fe_65_Co_35_-ZnO films by Wang et al. [9], which occurred in the several nanometer areas between FeCo and FeCo-SiO_2_ layers. As a result, the smaller anisotropy field, compared to the monolayer films, would move the resonant frequency to low frequency, reduce the coercivity, and improve the permeability which fits well with the experiment result.

## Conclusions

The FeCo-SiO_2_ monolayer films and FeCo/(FeCo)_0.63_(SiO_2_)_0.37_ multilayer films, with the same FeCo content 72 at %, were all elaborated on flexible substrates by magnetron sputtering system. In both kind of films, the FeCo metal particles are embedded in insulating SiO_2_matrices and presented polycrystalline structure. Because of the decrease of the anisotropy field by adding FeCo layer, the high-frequency permeability of FeCo/(FeCo)_0.63_(SiO_2_)_0.37_ multilayer films have a huge improvement. Specifically, the real and imaginary parts of permeability, more than the double value of monolayer films, are raised to 250 and 350, respectively. Meanwhile, the coercivity *H*_c_ is down to 10 Oe, and the resonant frequency of multilayer films is down to 2.3 GHz.

## Competing interests

The authors declare that they have no competing interests.

## Authors’ contributions

LZ carried out the study of the nanogranular films about high-frequency properties, participated in the statistical analysis, and drafted the manuscript. SQ carried out the performance measurement of the films and participated in the manuscript modification. XW carried out the data integration and statistical analysis. JX participated in the design and coordination of the study. LD conceived of the study. All authors read and approved the final manuscript.
